# Depression, anxiety and stress in Swedish midwives: A cross-sectional survey

**DOI:** 10.18332/ejm/124941

**Published:** 2020-07-27

**Authors:** Annika Båtsman, Hanna Fahlbeck, Ingegerd Hildingsson

**Affiliations:** 1 Department of Women’s and Children’s Health, Uppsala University, Uppsala, Sweden; 2 Department of Psychiatry, LARO, University Hospital of Umeå, Umeå, Sweden; 3 Uppsala University Hospital, Uppsala, Sweden; 4 Department of Nursing, Mid Sweden University, Sundsvall, Sweden

**Keywords:** depression, anxiety, stress, midwife

## Abstract

**INTRODUCTION:**

Midwives are exposed to emotional strain, which could affect their overall health. Lack of emotional well-being could be a reason for workforce attrition. The aim of the study was to investigate the prevalence of depressive symptoms, anxiety and stress among Swedish midwives in relation to background variables.

**METHODS:**

A random sample of 1000 midwives were asked to participate and complete a questionnaire. Participants completed the Depression, Anxiety and Stress Scale, Copenhagen Burnout Inventory and Quality of Life inventories together with demographic and work-related data.

**RESULTS:**

In all, 470 midwives responded to the questionnaire (48%). The prevalence of moderate/severe/very severe symptoms of depressive symptoms was 12%, anxiety 8.6%, and stress 7.2%. Midwives aged <40 years and those with <10 years work experience reported higher levels of depressive symptoms, anxiety and stress. The factors most strongly associated with symptoms of depression were personal burnout (AOR=12.26), client burnout (AOR=1.95) and quality of life (AOR=0.26) The factors most strongly associated with symptoms of anxiety were work burnout (AOR=2.53) and personal burnout (AOR=5.61). The factors most strongly associated with stress were personal burnout (AOR=3.90) and work burnout (AOR=3.58) and high quality of life (AOR=0.34).

**CONCLUSIONS:**

Swedish midwives experience symptoms of depression, anxiety and stress. Symptoms of burnout were associated with all aspects of mental health, while high quality of life was protective against these symptoms. These findings are relevant to consider in the work environment for Swedish midwives in order to reduce attrition rates.

## INTRODUCTION

Midwives in Sweden are the primary providers of maternity care, although they usually work in a highly specialised and largely fragmented way. There is a fast development of knowledge in healthcare, which sets high demands on midwives and midwifery education. Midwives require knowledge about sexual and reproductive health, the ability to inform and provide counseling as well as the skill to perform examinations and treatments. They are also expected to promote a healthy lifestyle and prevent poor health, contribute to research and education as well as lead and organise work^1^. In the beginning of the 2010s, work-related illness increased in Sweden, especially among women in women-dominated sectors. The work environment is believed to be of great significance in this increase^2^. In a recent meta-ethnographic study of 11 qualitative studies, the result showed that midwives questioned their professional choice and considered leaving the profession for an alternative career^3^.

The main reasons were that midwives were exposed to strain on a daily basis, which put pressure and stress on their overall health^3^. Similarly, a qualitative study showed that a heavy workload created feelings of anxiety among midwives in New Zealand^4^. Approximately 20% of the 1037 midwives in a cross-sectional study in Australia had moderate to severe symptoms of depression, anxiety and stress^5^, and more than 30% in a recent study from the UK^6^. A qualitative study among midwives in the UK showed that workplace and working conditions affected midwives’ mental health^7^. The workplace at times created situations in which the midwife experienced a loss of control. The different stressors included a heavy workload, shortage of staff, few opportunities to take breaks, extensive administrative work and stressed colleagues^7^. According to a cross-sectional study performed at a Croatian university hospital with 300 midwives and nurses in child healthcare, the majority experienced stress in relation to their work and the most stressing factor was insufficient work resources. Other stressors were unexpected situations, inadequate income, working night shift and increasing number of sick patients. Lack of communication with work management and flaws in the organisation were also reported as stressors^8^.

Poor emotional health could be of different kind. Depression is a common mental illness that is defined by a persistent lowered mood, difficulty in performing daily tasks as well as a reduced interest in usual activities. Lethargy, changes in appetite, anxiety, feelings of guilt, impaired concentration and hopelessness are also frequent symptoms of depression, sometimes accompanied by self-harm and suicidal thoughts^9^. Anxiety is an emotional state where tension and nervousness are often combined with physical reactions, such as increased heart rate and blood pressure, sweating, shaking and dizziness^10^. Workrelated stress is defined as a feeling of not living up to the expectations of the workplace, having little influence on the work situation and lacking support from colleagues and superiors. If this continues for a long time without the possibility of recuperation and the demands exceed the resources, the work environment becomes unhealthy^11^.

The prevalence of depression in Sweden varies depending on population, measuring instruments and diagnostic criteria^12-14^. Studies have shown that 16% of the Swedish population reported impaired mental well-being^14^. Signs of anxiety, and major worries were reported by 36% of the population and the level of stress was experienced as high or severe by 14%^14^. There is also evidence of the comorbidity between depression and anxiety^15^. The prevalence of impaired mental health in the Swedish population in 2016 was higher among women than men and decreased with increasing age^14^. The incidence of stress was also higher in women and decreased with increasing age^13^. The feeling of anxiety and worry were more common among women and young people^16^.

Other circumstances could also interfere with well-being and work attrition; one such factor is perceived quality of life. Quality of Life (QoL) is defined by an individual’s own estimation of well-being. It is a broad concept that is influenced in a complex way by the individual’s physical and mental health, personal values, social relationships and his/ her relation to the prominent aspects of the environment^17^. Midwives and nurses who described their mental quality of life as low were, to a greater extent, thinking about leaving their jobs. The worse they estimated their own health, the more they thought about leaving the profession^18^.

Burnout is another condition that could influence midwives work and well-being. Burnout is defined as a multidimensional syndrome with symptoms of emotional fatigue, empathy loss and a deterioration of work performance. Burnout arises as a result of a crisis in ones’ relation to work. Feelings of inadequacy and lack of confidence in the professional role are very common, as well as physical exhaustion with fatigue and lack of energy^19^.

A recently published cross-sectional study with 598 Norwegian midwives showed that the prevalence of burnout in relation to personal and work-related factors was around 20%^20^. The prevalence of total burnout among a smaller group of Australian midwives, measured with Copenhagen Burnout Inventory, was 30%. Approximately 50% of the participants indicated that they were experiencing personal and work-related burnout. The client-related burnout was considerably lower^21^. There is an ongoing discussion about burnout and its relation to depression and anxiety, e.g. if burnout is a condition itself or if it causes depression and anxiety or if it is a consequence of depressive symptoms^22-24^.

Several scientific studies have shown associations between burnout, age and years in the midwifery profession^20,25-27^, where younger midwives and those with shorter work experience more at risk. Creedy et al.^5^ as well as Hunter et al.^6^ found significant links between high levels of burnout, and high levels of depression, anxiety and stress among midwives. This was observed in relation to personal and work-related burnout in particular. Studies have also shown a correlation between burnout and depression in occupational groups other than midwives^24,28^. The aim of this study was to investigate the prevalence of symptoms of depression, anxiety and stress among Swedish midwives in relation to background variables, symptoms of burnout and quality of life.

## METHODS

### Design

A quantitative cross-sectional design was used. These Swedish data were collected as part of an international network established in 2011, entitled Work, Health and Emotional Life of Midwives (WHELM)^5,6,20,26,27,29^. WHELM is a network of researchers who willingly share their work so others could replicate it and benchmark against each other. Ethical approval is handled by each individual country/ project team and there is no joint protocol published by the network. Researches from Australia, New Zealand, Sweden, Norway, Germany, UK, and Canada are members of the network.

### Study participants

An invitation to participate in the study was sent to 1000 randomly selected midwives, of a total of 6500 midwives who were members of the Swedish Association of Midwives and under the age of 65 years. The distribution of the questionnaire was done during one month in 2012. The research team got a list of all members of the association, and from this list a random sample was selected, using a computer program. Thereafter, a questionnaire and letter of information were mailed to the selected members.

Those who chose to participate completed the survey and returned it in the supplied prepaid envelope. Those who did not respond received a reminder one month later and the Swedish Association of Midwives also published a reminder in their newsletter/member journal. Of the 1000 surveys that were sent out, eleven were excluded because the participants were not working as midwives, five were accidentally sent to inaccurate addresses, and six were deemed unusable because the midwives in question were not working in Sweden or because the surveys were returned empty, leaving 470/978 (48%) completed questionnaires. Detailed information about the recruitment is reported elsewhere^27^.

### Data collection

Data were collected using a questionnaire with background questions, workplace questions and several validated instruments. The original WHELM survey was developed in Australia, and some of the background questions were altered to suit the Swedish context. No changes were made to the validated instruments used in this study. This paper reports on findings from some of the instruments: the 21-item Depression, Anxiety and Stress Scale (DASS-21)^30^, the Copenhagen Burnout Inventory (CBI)^31^, and the Quality of Life Scale^32^.

The Depression, Anxiety and Stress Scale (DASS-21), has been translated and validated in Sweden in three samples comprising a total of 624 persons (295 university students, 192 patients in primary care and 156 patients undergoing an online treatment for stress and anxiety)^33^. DASS-21 has been shown to be reliable and easy to use. Furthermore, demographic variables have a very limited impact on the outcome^34^. DASS-21 is considered useful for clinical needs, as well as for researchers studying depression, anxiety and stress^35^. DASS-21 comprises three subscales: depressive symptoms, anxiety and stress^30^. Each subscale comprises seven items and the participants complete the scale on 4-point rating scales ranging from 0 = ’Never’ to 4 = ’Almost always’. The scores on each subscale are added and further classified into categories of severity (normal, mild, moderate, severe, and extremely severe). In the multivariate analysis those who scored ‘normal’ were compared with all other categories of severity.

Burnout was assessed using the Swedish version of the Copenhagen Burnout Inventory (CBI)^31^. The scale originated from the belief that the foundation of burnout is primarily fatigue and exhaustion, which was considered to be in line with the historical development of the concept. The CBI scale consists of 19 questions divided into three subscales: personal burnout (6 items), work burnout (7 items) and client burnout (6 items). For example, the questions could be: ‘How often do you feel emotionally exhausted?’, ‘Do you feel weary at the end of your workday?’ or ‘Do you experience difficulty working with women/families?’. All items are assessed on 5-point scales. CBI is usually dichotomised as 0–49 = ‘No burnout’, ≥50 = ‘burnout’^31^.

To measure quality of life, the Quality of Life Scale (QoLS) was used^32^. The scale contains 16 items assessed on a scale from 1–7, with 1 being ‘terribly unsatisfied’ and 7 being ‘very satisfied’, with a maximum score of 112. The questions concerned the participants’ health, social interactions and close relationships. The items are summed to generate a total score. The score was further dichotomised into High QoL (>75th percentile) = 1 versus Low QoL (<75th percentile) = 0. The choice of using the cutoff point of 75th percentile was the ability to use the scale as a categorical variable rather than a continuous, in order to investigate the impact of high QoL.

### Process and analysis

The completed questionnaires were processed and analysed using the Statistical Package for Social Services (SPSS) version 24. Independent t-tests or ANOVA were performed to compare differences in the mean values and the background factors. Correlations between the instruments were explored using Spearman’s correlation. Reliability was measured with Cronbach alpha for both DASS-21 as a whole and the subscales depression, anxiety and stress and also for CBI and QoL. The Cronbach alpha coefficient was 0.921 for DASS-21. Cronbach alpha for the separate subscales was 0.879 for depressive symptoms, 0.777 for anxiety, and 0.856 for stress. Cronbach alpha values for the Copenhagen Burnout Inventory were 0.90 for the total score, 0.87 for personal burnout, 0.93 for work burnout, and 0.81 for client burnout. Cronbach alpha for QoL was 0.88. The strength of the relationships was interpreted based on Cohen’s guidelines (small = 0.1, medium = 0.3, large = 0.5)^36^.

DASS-21 subscales were used as continuous variables when calculating difference in mean values but were also presented in categories, based on the user’s manual: normal, mild, moderate, severe and very severe^30^. Finally, in order to reveal what factors were most strongly important in explaining any symptoms of depression, anxiety and stress, a logistic regression analysis was performed. We compared midwives with no symptoms (0) to those with mild/moderate/severe/very severe symptoms (1). Statistically significant variables (background data, burnout, and QoL) were entered into the model and presented as adjusted odds ratios with a 95% confidence interval.

The study underwent an ethical review by the Regional Research ethics committee in Umeå, Sweden (DNR 2012314-31Ö). Confidentiality was ensured, as no personal data were collected in the survey. The participants consented by returning the questionnaire, and they were informed that they could opt out of the study at any time.

## RESULTS

Of the 470 midwives who completed the questionnaire, all female, the majority (73.6%) were over 40 years of age, had their own children (89.5%) and were living with a partner (84.5%). They had a long working experience with 42% more than 20 years, and 24.1% who had been working at their current workplace between 10–20 years. The most common way of work distribution was daytime only (44.7%) and two-shifts (25.2%). In total, 61.2% were working fulltime and the most common weekly working hours were 32. More than half of the midwives (52.9%) worked in more than one area and the three most common workplaces were the antenatal clinic (30.1%), labour ward (41.1%) and postnatal ward (21.6%) (Table 1).

**Table 1:** Sociodemographic characteristics of participating midwives (N=470)

*Characteristics*	*n (%)*
**Age** (years)	
25–39	122 (26)
40–60	348 (74)
**Own children**	
No	394 (85)
Yes	72 (15)
**Civil status**	
Living with a partner	420 (90)
Not living with a partner	49 (10)
**Work experience as midwife** (years)	
<10	197 (42)
≥10	273 (58)
**Work distribution**	
Daytime only	206 (45)
Two-shift	116 (25)
Three-shift	84 (18)
Nighttime only	54 (12)
**Working hours**	
Full-time	284 (61)
Part-time	180 (39)
**Work in more than one area**	
No	236 (53)
Yes	210 (47)

Table 2 presents the mean values and levels of symptoms. The majority of midwives reported no symptoms of depression (76.8%), anxiety (88%) or stress (83.3%).

**Table 2:** Mean values and levels of symptoms of depression, anxiety and stress

	*Symptoms of depression n (%)*	*Symptoms of anxiety n (%)*	*Symptoms of stress n (%)*
**Mean ± SD**	5.58 ± 6.35	2.82 ± 4.34	8.36 ± 6.84
**Levels of symptoms**			
Normal	351 (76.8)	402 (88.0)	383 (83.3)
Mild	51 (11.2)	16 (3.5)	44 (9.6)
Moderate	43 (9.4)	24 (5.3)	23 (5.0)
Severe	5 (1.1)	9 (2.0)	9 (2.0)
Very severe	7 (1.5)	6 (1.3)	1 (0.2)

### Social background factors

All three subscales in DASS-21 were compared with the background variables (Table 3). There were significant differences between midwives of different ages. Midwives younger than 40 years reported higher symptoms of depression, anxiety and stress compared with their older colleagues. Similarly, the length of work experience was associated with symptoms of anxiety, which affected midwives with shorter work experience who scored higher in anxiety than midwives with longer work experience (p=0.043). Symptoms of stress were also more common in midwives who had work experience of fewer than 10 years, compared to their more experienced counterparts (p=0.003). The other variables showed no statistical significance.

**Table 3:** Symptoms of depression, anxiety and stress in relation to background factors^a^

	*Symptoms of depression mean (SD)*	*Symptoms of anxiety mean (SD)*	*Symptoms of stress mean (SD)*
**Age** (years)			
25–39	6.81 (7.58)*	3.86 (5.44)*	10.74 (7.39)***
40–60	5.19 (5.80)	2.47 (3.85)	7.54 (6.46)
**Own children**			
No	7.27 (8.38)	3.67 (.71)	9.67 (8.1)
Yes	5.4 (6.03)	2.72 (4.16)	8.22 (6.68)
**Civil status**			
Single	5.94 (7.14)	2.97 (3.94)	7.51 (6.43)
Living with a partner	5.53 (6.21)	2.80 (4.44)	8.53 (6.94)
**Work experience as midwife** (years)			
<10	6.36 (7.37)	3.48 (5.26)*	9.72 (7.11)**
≥10	5.22 (5.75)	2.50 (3.80)	7.69 (6.36)
**Work distribution**			
Daytime only	5.27 (6.627)	2.57 (4.15)	8.00 (6.63)
Two-shift	5.86 (6.90)	3.60 (4.89)	9.23 (7.78)
Three-shift	5.81 (5.61)	2.96 (4.83)	8.72 (6.45)
Nighttime only	5.93 (6.62)	2.11 (2.87)	7.47 (6.20)
**Working hours**			
Full-time	5.15 (5.72)	2.66 (4.10)	8.19 (6.51)
Part-time	6.23 (7.17)	3.10 (4.73)	8.64 (7.76)
**Hours worked per week**			
8–32	6.19 (6.97)	3.13 (4.79)	8.76 (7.20)
36–40	5.17 (5.81)	2.63 (4.03)	8.10 (6.62)
**Work in more than one area**			
No	5.72 (6.66)	2.91 (4.65)	8.57 (7.32)
Yes	5.52 (6.09)		

a t-tests or ANOVA.

* p<0.05.

** p<0.01.

*** p<0.001.

### Correlation between DASS-21, burnout and quality of life

All three subscales of DASS-21 were significantly correlated with the three subscales of burnout, with the highest correlations found in personal and work-related burnout (Table 4). In addition, the Quality of Life scale was negatively correlated with the three subscales of DASS-21. There were strong, statistically significant correlations between the CBI Work subscale and all three subscales of DASS-21 with all Spearman correlation coefficient 0.55 to 0.70. For the CBI Work subscale, the correlations were large for Depression and Stress subscale and moderate for the Anxiety subscale. The Client subscale of CBI yielded only small correlation coefficients. The QoL resulted in a large correlation coefficient with the Depression subscale of DASS-32, but small for Anxiety and Stress.

**Table 4:** Spearman’s correlation coefficients (ρ) between DASS-21, CBI, and QoL

	*Depression*	*Anxiety*	*Stress*
CBI Personal burnout	0.66	0.49	0.60
			
CBI Work burnout	0.70	0.55	0.67
			
CBI Client burnout	0.38	0.27	0.38
			
Quality of life	-0.55	-0.35	-0.47
	2.70 (4.06)	8.11 (6.45)	

Table 5 shows the mean differences in midwives with and without burnout symptoms as well as those with and without high quality of life. Burnout symptoms created statistically significant differences for all the subscales of DASS-21, while midwives with high quality of life (75th percentile) were less likely to report symptoms of depression, anxiety and stress.

**Table 5:** Burnout and quality of life in relation to depressive symptoms, anxiety, and stress^a^

	*Symptoms of depression mean (SD)*	*Symptoms of anxiety mean (SD)*	*Symptoms of stress mean (SD)*
**Burnout**			
Personal burnout	10.26 (7.24)***	4.97 (5.62)***	12.69 (7.13)***
No personal burnout	2.62 (3.16)	1.42 (2.43)	5.47 (4.87)
Work burnout	9.32 (7.51)***	4.93 (5.67)***	12.77 (7.13)***
No work burnout	3.66 (4.59)	1.69 (2.89)	6.00 (5.40)
Client burnout	10.88 (8.05)***	5.13 (6.39)***	12.47 (7.32)***
No client burnout	4.64 (5.53)	2.42 (3.77)	7.56 (6.52)
**Quality of Life**			
Low (<75 Percentile)	6.97 (6.80)***	3.41 (4.77)***	9.79 (6.97)***
High (>75 Percentile)	2.21 (3.03)	1.34 (2.51)	4.78 (4.97)

a t-test was used in the analysis.

*** p<0.001.

### Multivariate analysis

Finally, the results from the multivariate analysis showed that Personal burnout (AOR=12.26; 95% CI: 6.05–24.25), and Client burnout (AOR=1.95; 95% CI: 1.07–3.72) increased the risk for any depressive symptoms, while high quality of life decreased the likelihood (AOR=0.26; 95% CI: 0.10– 0.62). For anxiety symptoms Personal burnout (AOR=5.61; 95% CI: 2.27–13.86) and Work burnout (AOR=2.53; 95% CI: 1.19–5.36) increased the risk. For stress symptoms Personal burnout (AOR=3.90; 95% CI: 1.85–8.21) and Work burnout (AOR=3.58; 95% CI: 1.83–7.01) increased the risk, while high QoL was protective (AOR=0.34; 95% CI: 0.13– 0.88).

## DISCUSSION

The main results of this study were that any symptoms of depression, anxiety and stress in Swedish midwives were quite common. The age of the respondent midwife and the number of years in the profession affected the results, but the work situation and other sociodemographic characteristics had a relatively small impact on the variables. The most important factors, however, were symptoms of burnout. The results also showed that high quality of life was protective in some subscales.

The prevalence of symptoms of moderate to severe depression, anxiety or stress were between 7% and 12%, which is lower than in a study of midwives in Australia^5^ as well as in the UK^6^. Compared to information about mental health issues in the Swedish population^12,13,16^, the prevalence of symptoms of moderate/severe/very severe depression, anxiety and stress was lower in the present work then in the general population. Comparing prevalence always poses a risk if the measurements are made with different instruments. The prevalence of symptoms in the present work can, above all, be compared only with prevalence measured according to DASS-21. Although the prevalence of moderate/severe/very severe symptoms of depression, anxiety and stress were lower than previously reported in international midwifery studies^5,6,20^, the background factors associated with high levels of DASS-21 were quite similar to other studies, such as short working experience and young age.

Burnout was seen as a factor that explained the presence of symptoms of depression, anxiety and stress and had the highest significance for the results in this study. These findings were strengthened by previous research showing similar associations among midwives^5,6^ and other professions^22,24,28,37-40^. The connection between burnout and workplace factors has also been shown in previous research^20^ and studies have shown that midwives’ mental health was affected by the working conditions and working environment^7^, organisational aspects^7,8,41^, and lack of resources^8,27,39^. Since burnout is such an important factor in regard to symptoms of depression, anxiety and stress, factors associated with symptoms of burnout should be taken into account when identifying and working with midwives’ emotional health. The present study demonstrated that the level of symptoms of depression, anxiety and stress on average was higher in midwives with symptoms of burnout, compared to those who did not experience burnout. The prevalence and associated factors of burnout symptoms among the same group of midwives has been published in a previous study^27^ but did not take into account the constructs of DASS-21. That study showed that symptoms of burnout were more common in midwives who were younger and had worked fewer years in the profession. One-third had thought about leaving, mainly because of the situation at the workplace, with lack of resources and staff. The results of the present study, which demonstrate the relationship between symptoms of burnout and symptoms of depression, anxiety and stress, highlight the need to work with workplace factors to counteract burnout and protect midwives from poor mental health.

High quality of life was negatively associated with symptoms of depression, anxiety and stress in the results in the present study. There is a lack of studies focusing on midwives and their quality of life in relation to emotional symptoms. The majority of previous studies combine results from midwives and nurses, such as a previously published study with Greek nurses that showed that work contributed to the experience of quality of life^40^. Quality of life is interconnected with various circumstances in life, such as work satisfaction^18^, and symptoms of poor mental health^42^, as well as depression^22,43^.

The results of the present study show that age matters. Younger midwives experienced more symptoms of depression, anxiety and stress compared to their older colleagues. This corresponds with multiple studies showing this association among midwives and nurses when burnout, fatigue and work satisfaction has been studied. Henrikssen and Lukasse^20^ found that age 60 years or more was a factor resulting in lower incidence of work-related burnout among midwives. The fact that the midwives in the present work reported lower levels of depressive symptoms and symptoms of anxiety and stress compared with The Public Health Agency in Sweden measurements might be explained by the fact that the majority of the participating midwives were aged >40 years. The fact that older age was associated with a lower prevalence of symptoms of depression, anxiety and stress was also confirmed by Johansson et al.^12^ and the Public Health Agency in Sweden^13,14,16^. Similarly, in a study by Perry et al.^18^, the authors reported that midwives and nurses aged >45 years estimated their mental quality of life higher than their younger colleagues, which was similar to the result that the midwives aged >40 years in this study had fewer symptoms of depressions, anxiety and stress compared to their younger colleagues. These results do not indicate a causal relationship, but highlight the complexity of the balance between work life and mental health. The quality of life of the midwife, its possible relationship with job satisfaction and its importance for a mentally healthy working life should be further investigated. The current study also showed that midwives who had worked fewer years in the profession felt more symptoms of stress than their more experienced colleagues. According to McGarath et al.^40^, stress could be relieved with the advice and support of more senior colleagues. Previous research indicates that the midwifery culture involves exposure to emotionally challenged situations^3,25,43^ and higher demands on midwives, according to the Swedish National Board of Health and Welfare^1^. This fact makes the less experienced midwives a vulnerable group. A stressful work environment requires quick decisions and a staff with high skills. The stress to which midwives are exposed on a daily basis is assumed to be harder for younger people in the profession.

In the present study the participants worked in different areas of midwifery. The majority worked in antenatal, intrapartum and postpartum care, but Swedish midwives also work in areas such as sexual health, gynaecology and youth clinics. Eadie and Sheridan^4^ found that anxiety in midwives was common if they felt that they lacked skills to work with severely ill pregnant women. A reasonable assumption was that increased skills came over the years, which could match the results in the present study, which showed that midwives with shorter work histories experienced more anxiety. According to Elmir et al.^3^, a possible way to alleviate the sense of incompetence in midwives was organised support and inter-professional cooperation.

### Limitations and strengths

The study is limited because of the relatively low response rate. During the period of data collection there was an extensive debate about burnout among Swedish midwives and many left their work. This could be one reason for the low response rate. Another explanation could be that non-responders were affected by burnout or symptoms of depression anxiety and stress and therefore found it difficult to be exposed to questions in that area. On the other hand, it might be possible that only midwives with symptoms of depression, anxiety or stress were more interested in completing the survey. There is no way to determine if the sample was representative of all midwives in Sweden, which undermines the results about prevalence, but age and work distribution were similar to those reported in national statistics^44^.

Data were collected in 2012, a fact that should be noticed, as it is possible that the work environment might have changed over the years. The number of midwives leaving the profession is said to be increasing, and Swedish decision makers and politicians have added funds to improve the situation in labour wards and obstetric care. The funding has been used in many ways within the Swedish regions. One example is that more midwives have been employed in some hospitals, and in other regions trainee programs for newly graduated midwives have commenced^45^. The effect of these initiatives on midwives’ experiences are, however, largely unknown. National statistics show that midwives working in hospital-based care have decreased from 66.5% in 2012 to 64% in 2017. Those working in primary healthcare increased from 25% to 27% during the same time span^46^. From the present survey we do not know how many midwives have actually left the profession, which is a limitation. A previous publication from the Swedish study^27^ showed than one in three midwives were ‘thinking of leaving the profession’. Other ways of dealing with emotional stress in healthcare providers, mainly those who work in labour wards has been suggested. Resilience training to prevent the development severe symptoms of anxiety, that could lead to PTSD, is one such suggestion^47^. Nevertheless, as the associated factors are similar to other studies, there are reasons to believe that the data still are stable and trustworthy. Despite the age of the data, we believe that this study could serve as a normative measure for further comparisons of DASS-21.

Another limitation is that the questionnaire covered many instruments and questions of which DASS-21 was only a small part. The internal missing values were low overall (<3%), which was considered negligible. The large number of questions provided a good opportunity for research but could be experienced by the respondent as too extensive.

The strength of the study was that the sample was randomly selected from midwives who were members of the Swedish Midwives Association. The questionnaire is believed to have reached a large selection of Swedish midwives since approximately 91% of the registered midwives were members of the Association in 2012.

In the univariate analysis, moderate/severe/very severe symptoms of depression, anxiety and stress were merged and grouped and compared with those without or mild symptoms, based on the calculation recommended by the creators of DASS-21^30^. It could be difficult to compare the prevalence with research where depression, anxiety and stress are clinically diagnosed or where the frequency is based on DSM-criteria. It can be discussed whether midwives with mild symptoms of depression, anxiety and stress should have belonged to the group that was free of ailment or the group where more severe symptoms were present. However, according to the Swedish Public Health Institute^14^, even mild symptoms can cause suffering even though they do not need to reflect a psychiatric diagnosis. In the multivariate analysis, however, participants with any levels of symptoms were compared with those with no symptoms. This was a better solution and created more comparable groups, otherwise the confidence intervals of the odds ratios were very wide, indicating lack of precision.

Validated instruments were used in the study. However, the CBI and DASS-21 were only used on midwives in a few studies, but the instruments were considered adequate for the chosen purpose and the factors associated with depressive symptoms, anxiety and stress quite similar^5,6^. The reliability was high according to Cronbach alpha for both DASS-21 overall and for the respective subscales measuring symptoms of depression, anxiety and stress.

Several studies highlight the importance to focus on midwives’ health and wellbeing in order to maintain the workforce. Risk factors linked to the workplace could and should be reduced. An opportunity for professional development and organised support from work managers and older experienced colleagues might relieve the experience of depression, anxiety and stress symptoms among midwives. Further research is important nationally and internationally to continue the investigation of these subjects, and in so doing find the solutions to the problems faced in the field.

## CONCLUSIONS

The prevalence of moderate to severe symptoms of depression, anxiety and stress in this sample of midwives in Sweden were between 7–12%. Younger midwives and midwives with fewer years in the profession were affected to a greater extent than their older and more experienced colleagues. There were strong connections between quality of life and respective symptoms of burnout and depression, anxiety and stress but the social background of the different midwives was of less consequence. The increase in workrelated poor health and burnout can be classified as a public health problem and could further affect women’s and children’s health and well-being. In addition to public health interventions, changes in the workplace are required to strengthen the quality of life and reduce the incidences of depression, anxiety and stress symptoms, and burnout, thereby promoting midwives to remain in the profession throughout their professional lives.
